# Using social media user attributes to understand human–environment interactions at urban parks

**DOI:** 10.1038/s41598-020-57864-4

**Published:** 2020-01-21

**Authors:** Xiao Ping Song, Daniel R. Richards, Puay Yok Tan

**Affiliations:** 1 Department of Architecture, National University of Singapore, 4 Architecture Drive, Singapore, 117566 Singapore; 2 Future Cities Laboratory, ETH Zurich, Singapore-ETH Centre, 1 Create Way, CREATE Tower, #06-01, Singapore, 138602 Singapore

**Keywords:** Ecosystem services, Urban ecology, Psychology and behaviour, Sustainability

## Abstract

Urban parks and green spaces are among the few places where city dwellers can have regular contact with nature and engage in outdoor recreation. Social media data provide opportunities to understand such human–environment interactions. While studies have demonstrated that geo-located photographs are useful indicators of recreation across different spaces, recreation behaviour also varies between different groups of people. Our study used social media to assess behavioural patterns across different groups of park users in tropical Singapore. 4,674 users were grouped based on the location and content of their photographs on the Flickr platform. We analysed how these groups varied spatially in the parks they visited, as well as in their photography behaviour. Over 250,000 photographs were analysed, including those uploaded and favourited by users, and all photographs taken at city parks. There were significant differences in the number and types of park photographs between tourists and locals, and between user-group axes formed from users’ photograph content. Spatial mapping of different user groups showed distinct patterns in the parks they were attracted to. Future work should consider such variability both within and between data sources, to provide a more context-dependent understanding of human–environment interactions and preferences for outdoor recreation.

## Introduction

Outdoor recreation is an important component of leisure and tourism^1^ and provides opportunities for people to experience nature^2^. Visits to protected areas and national parks are a major contributor to recreational experiences^3,4^, and generate approximately US $600 billion a year in direct expenditure within local economies worldwide^5^. Within urban areas, parks and green spaces are among the few places where city dwellers can have regular contact with nature^6^. A growing body of literature has shown that these urban green spaces provide a host of measurable benefits to city dwellers, such as improved physical health, cognitive performance and psychological well-being^7–9^. As urban populations continue to grow and competition for land resources become more intense^10^, the planning and management of recreational resources in cities are becoming increasingly important^11^.

Information on the use of urban parks is commonly lacking, particularly at the scale of entire cities^12,13^. In the past few years, there has been rapid growth in the number of studies using geo-located social media data to assess spatial patterns of recreation and human–environment interactions^14^. Information from these sources of online ‘big data’ provide wide coverage across time and space, in contrast to local surveys that are often costly and resource-intensive^3^. Although social media data carry inherent uncertainty surrounding selection bias and data quality^15^, strong links between geo-tagged photographs and empirical visitor counts have been observed at parks and outdoor attractions^16^. Alongside conventional survey techniques, city-wide analyses of social media data have helped uncover potential drivers of park visits^17^.

Social media have previously been used to assess patterns of recreation, both within and across different places^4,18–20^. Past research has typically analysed the entire population of social media users as a whole, thus assuming that all people are uniform in their preferences^4,17,18,21^. However, urban parks are used by many different types of people who have different motivations and constraints, so it is also important to analyse varying behavioural patterns between different groups. The interactions that people have with the environment are shaped by their experiences, and there is growing demand for a personalised approach that takes into account human variability when examining human–nature interactions^22^. Face-to-face and online surveys of park use have previously found key differences in recreational behaviour between people, influenced by factors such as sex, age, ethnicity, pet ownership and income level^23–25^. However, such data are typically not available in studies relying on social media.

In addition to their use in understanding the popularity of outdoor spaces, social media data hold a wealth of information on the activities and interests^26^ of people, as well as their geographical range^27^. The photographs that people capture can reflect their interests, aesthetic values, sentimental attachment and emotional state at a particular time and place^28,29^, and a growing suite of approaches to interpret photograph content offers new ways to examine the place-based experiences of social media users^19–21,30,31^. Furthermore, people also share other kinds of photographs related to their personal interests^32^, and engage with other users by ‘liking’ or sharing their content^33,34^. In online advertising, the photograph content and activity of users have been used to identify groups of people with similar interests^32,34^. Similarly, the spatial information inherent in geo-located photographs have been used to group users according to their geographical origin^3,14,16,20,35^. Using the location and content of photographs that users upload and show appreciation for, there is potential to differentiate between multiple groups of park users, and to analyse differences in their recreation behaviour at urban parks.

In this study, we classified park users in tropical Singapore using photograph data within social media profiles. We examined the city-wide variation in their recreation behaviour at public parks, based on their photography behaviour at these locations. Our study assumes that the act of sharing constitutes some measure of the photographer’s use of the location, as well as individual preference for the depicted subject matter^29^. The study objectives were to:

1. Examine how the location and content of photographs in social media profiles can distinguish between different types of park users.
2. Analyse how different groups of users vary in the frequency and spatial distribution of park use.
3. Analyse how different groups of users vary in the types of photographs they capture at parks.

## Results

### Data extraction

**Figure 1:**
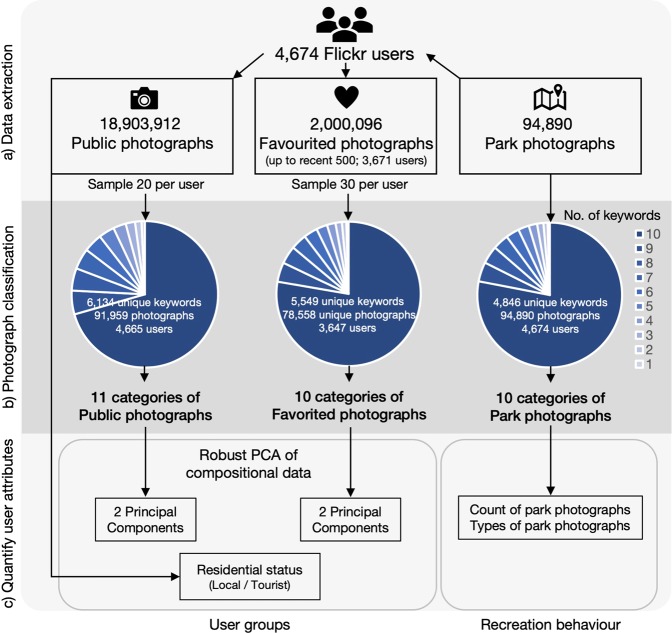
Social media data workflow. The study analysed recreation behaviour at urban parks between different groups of people, using photograph data on social media profiles.

The photograph-sharing platform Flickr was used owing to its open format and accessibility of data. The Flickr Application Programming Interface (API) was used to extract 94,890 photographs geo-located within parks in Singapore, uploaded by 4,674 users between February 2004 to March 2018 (Fig. 1a). The Google Cloud Vision API was used to interpret photograph content, and 4,846 unique keywords were generated from park photographs. Random subsamples of each park users’ public uploads produced 91,959 photographs with 6,134 unique keywords, and random subsamples of users’ favourited photographs produced 78,558 unique photographs with 5,549 unique keywords (Fig. 1b).

### Photograph classification

**Figure 2:**
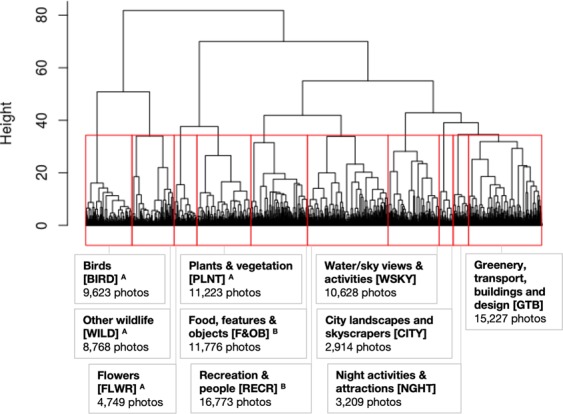
Ten categories of 94,890 park photographs after hierarchical clustering. The abbreviation for each category name is shown in square brackets. Superscripts denote the categories aggregated for regression analyses: ^A^NATURE; ^B^RECREATION. Categories of public and favourited photographs are in the Supplementary Information (Figs. S5 and S6; details on cluster analyses and resulting categories in Figs. S1 to S4).

Following the hierarchical clustering method proposed by Richards and Tunçer^21^, photographs were classified into content-type groups based on keyword labels generated by Google Cloud Vision (Figs. 1b and 2). The similarity between each unique pair of photographs was calculated based on the number of keywords that they share. This avoids subjective interpretation often associated with manual classification, and allowed photographs to be classified into discrete categories via hierarchical clustering, despite the presence of overlapping content.

Hierarchical clustering of users’ public, favourited and park photographs produced 11, 10 and 10 categories, respectively (Fig. 1b). The ten categories of park photographs were: Birds (BIRD); Other wildlife (WILD); Flowers (FLWR); Plants and vegetation (PLNT); Food, features and objects (F&OB); Recreation and people (RECR); Water or sky views and activities (WSKY); City landscapes and skyscrapers (CITY); Night activities and attractions (NGHT); Greenery, transport, buildings and design (GTB) (Fig. 2; see Supplementary Figs. S5 and S6 for public and favourited photographs). The overall classification accuracy for park, public and favourited photographs ranged from 65.5–74.5%, and the weighted Kappa value ranged from 0.68–0.78 (Supplementary Tables S1 to S3), indicating a relatively high level of agreement between automated and manual classification^36^. Results from the confusion matrices were used to identify photograph categories that had a high chance of mutual misclassification (i.e. tended to overlap), as well as those with a broad mixture of miscellaneous content (details in Supplementary Tables S1–S3). Related categories were then aggregated to improve the classification accuracy, as well as the interpretability of subsequent analyses (details for public and favourited photographs in Supplementary Figs. S5 and S6).

### Formation of user groups

Park user groups were derived from the photograph data available on Flickr users’ public profiles. User groups were based on (1) residential status, as well as the content of their (2) publicly uploaded photographs and (3) photographs that they showed appreciation for (i.e. ‘favouriting’ or ‘liking’) (Fig. 1c). Users’ residential status were assigned based on the country listed on their online profiles. Otherwise, it was defined as the country where most of the randomly-sampled public photographs were taken at (Fig. 1c). 1,916 locals and 2,758 tourists uploaded photographs at parks in Singapore.

**Figure 3:**
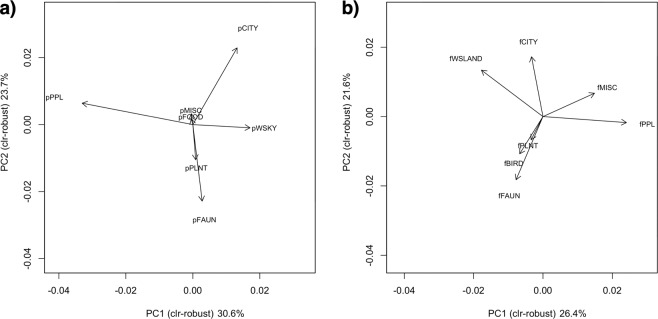
Loading plots for robust principal component analysis of park users’ (**a**) public and (**b**) favourited photographs. Details on photograph categories are in the Supplementary Information (Figs. S5 and S6; Tables S2 and S3).

The axes of content within users’ public and favourited photographs were formed by performing robust principal component analyses (PCA) on photograph categories within user profiles (Fig. 1c). The factor loadings of the principal components (PC) were used to examine the types of photographs with the highest contribution to each PC (Fig. 3), and to assign a qualitative name to each PC to aid interpretation. The first user axis differentiated between users whose public profiles contained more photographs of landscapes (pWSKY, CITY) against users whose profiles contained more photographs of people (pPPL) (PC1, Fig. 3a). Similarly, those who favourited photographs of landscapes (fWSLAND, fCITY) were less likely to do so for photographs of people (fPPL) (PC1; Fig. 3b). The second user axis differentiated between users whose public profiles contained more photographs of wildlife (pFAUN, pPLNT) from those who uploaded more photographs of the city (pCITY) (PC2, Fig. 3a). A similar axis was found in the favourited photographs; people who favourited more photographs of wildlife (fFAUN, fBIRD, fPLNT) were less likely to favourite photographs of the city (fCITY) (PC2, Fig. 3b). The first two PCs captured 54.3% and 48.0% of variance in the composition of users’ public and favourited photographs, respectively (Fig. 3). Although the patterns were broadly similar between public and favourited photographs, their correlation was fairly weak; Pearson’s correlation between the PC1s (hereafter referred to as the ‘Landscapes–People’ axes) was 0.33 (P < 0.001), and correlation between the PC2s (hereafter referred to as the ‘Wildlife–City’ axes) was 0.36 (P < 0.001). Therefore, all four variables were used as predictors for subsequent regression analyses.

### User variation in park photography

**Figure 4:**
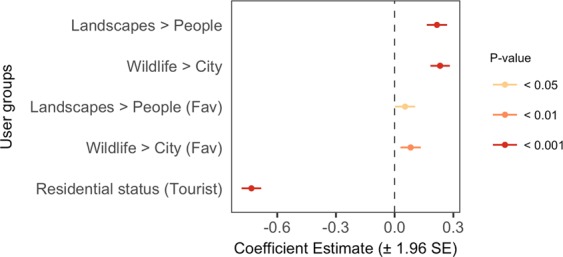
Coefficient plot showing the user groups that affect the count of park photographs in social media profiles. Multiple regression was performed using the negative binomial model (n = 3,616). Users were grouped based on the content of their public and favourited (Fav) photographs (principal component axes), as well as their residential status (binary variable).

Negative binomial regression was used to examine the effect of different user groups on the count of park photographs (Figs. 1c and 4). Although there were more tourists among the 4,674 park users, the number of photographs they captured at parks was fewer compared to locals (Fig. 4). We found that park photography was higher among users that uploaded more photographs of ‘Landscapes’ than ‘People’, and more of ‘Wildlife’ than the ‘City’; similar trends were observed among favourited photographs, though the effects were slightly weaker (Fig. 4).

**Figure 5:**
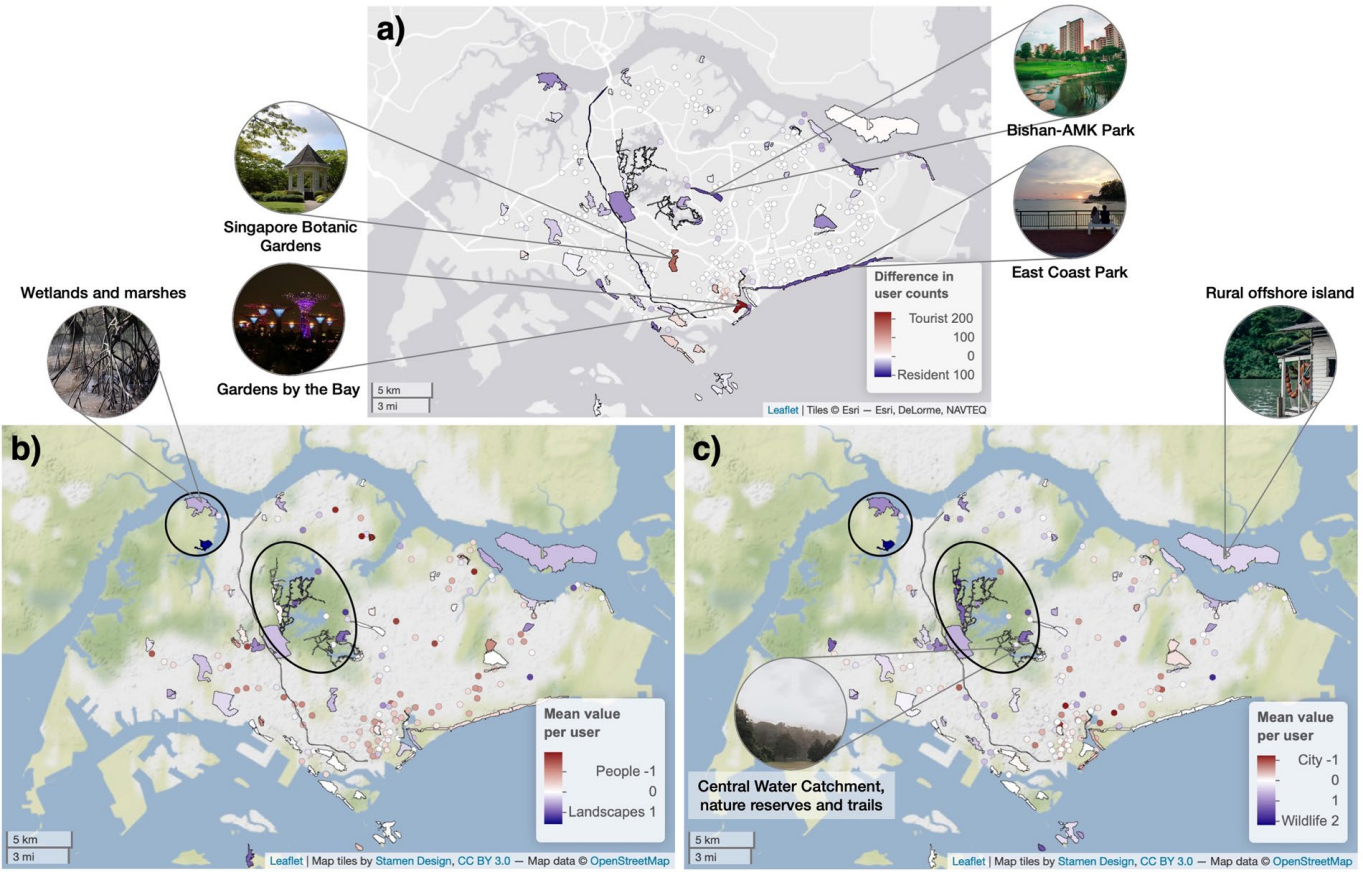
Variation across parks in Singapore according to the kinds of social media users they attract. User groups were based on (**a**) residential status, as well as the content of favourited photographs using the principal component axes (**b**) Landscapes–People and (**c**) Wildlife–City. Parks less than 0.1 km^2^ are shown as circles. Maps based on users’ uploaded photographs are in Supplementary Fig. S10. Data sources for base maps: Esri, DeLorme, NAVTEQ; Stamen Design; OpenStreetMap.

Visits by different groups of users were mapped spatially across parks in Singapore (Fig. 5). Major tourist attractions such as Gardens by the Bay and the Singapore Botanic Gardens attracted visits from many more tourists, while regional parks such as East Coast Park and Bishan-Ang Mo Kio Park were more popular among locals (Fig. 5a). Distinct spatial patterns in park users’ visit locations were observed based on their appreciation (i.e. favouriting) of photographs online. For example, parks with more natural landscapes (i.e. forests, nature reserves, rural areas) tended to attract users who favourited more photographs of ‘Landscapes’ than ‘People’ (Fig. 5b), and more of ‘Wildlife’ than the ‘City’ (Fig. 5c). A similar pattern was observed for users’ publicly uploaded photographs, but only for the user-group axis ‘Wildlife–City’ (Fig. 5c and Supplementary Fig. S10b); park visits based on the axis ‘Landscapes–People’ were less consistent between public and favourited photographs (Fig. 5b and Supplementary Fig. S10a).

### User variation in their types of park photographs

**Figure 6:**
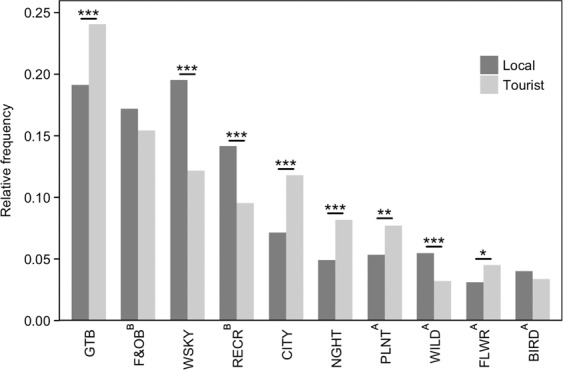
Types of park photographs captured by 4,674 users grouped according to their residential status. One photograph was sampled per user, and the mean frequency across 50 samples was calculated for each category (sensitivity analysis in Supplementary Fig. S9). Asterisks (*) indicate significant differences based on two-tailed Z-tests (*P < 0.05; **P < 0.01; ***P < 0.001). Superscripts denote the categories aggregated for regression analyses: A NATURE; B RECREATION. The category GTB contained a broad mixture of miscellaneous content and was excluded from regression analyses.

**Figure 7:**
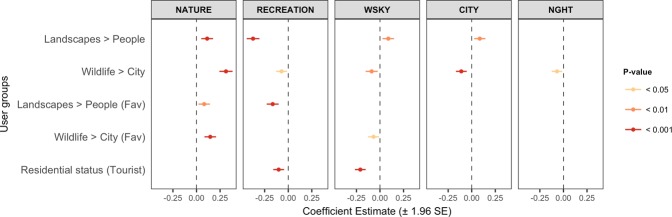
Coefficient plots showing the user groups that affect the types of park photographs in social media profiles (n = 1,177). Users were grouped based on the content of their public and favourited (Fav) photographs (principal component axes), as well as their residential status (binary variable).

Among the 4,674 park users, photographs of ‘recreation’, ‘water/sky views and activities’ and the ‘city’ were more frequently uploaded compared to those of ‘nature’ (Fig. 6). Chi-squared comparisons show that locals took more photographs of ‘water/sky views and activities’, ‘recreation’ and ‘wildlife’ (WSKY: *Z* = 47.5, P < 0.001; RECR: *Z* = 23.7, P < 0.001; WILD: *Z* = 14.7, P < 0.001) (Fig. 6), while tourists took more photographs of the ‘city’ and ‘night’ life, as well as ‘plants’ and ‘flowers’ (CITY: *Z* = 27.8, P < 0.001; NIGHT: *Z* = 18.8, P < 0. < 0.001; PLNT: *Z* = 10.1, P < 0.01; FLWR: *Z* = 6.2, P < 0.05) (Fig. 6). The relationships between each user group and various types of park photographs were examined (Figs. 1c and 7). Five categories of park photographs (Figs. 2 and 6) were analysed as a composition variable using Dirichlet regression^37^. Results show that photographs of ‘recreation’ and ‘water/sky view and activities’ at parks were significantly lower among tourists (Fig. 7, panel 2 and 3; Fig. 6). Park photographs of ‘nature’, ‘recreation’ and the ‘water/sky view and activities’ varied significantly between users grouped according to photograph content in their profiles (Fig. 7, panel 1–3). The relationships between user groups and park photographs of ‘nature’ (Fig. 7, panel 1) were very similar to those observed for park photograph counts (Fig. 4). These trends were reversed for park photographs of ‘recreation’ (Fig. 7, panel 2). Park photographs of ‘water/sky view and activities’ and ‘cities’ (Fig. 7, panel 3 and 4) tended to be higher among users who took more photographs of ‘Landscapes’ than ‘People’, and lower among users who took more photographs of ‘Wildlife’ than the ‘City’. Finally, users who took more park photographs at ‘night’ were more likely to upload photographs of the ‘City’ than ‘Wildlife’ (Fig. 7, panel 5).

## Discussion

Information about public perception and use of parks has played a role in park management and planning over the past few decades^38^. More recently, geo-located social media data has been used to assess patterns of recreation both within and across different places. These include the popularity^4,17^, use^19,20^ and aesthetic value^18,39^ of parks. In addition to quantifying these place-based experiences, identifying consistent behavioural patterns between groups of people can help enhance our understanding of human–environment interactions and preferences for outdoor recreation^11^. In this study, we extend this research approach and demonstrate the use of social media data to capture variation in the people who visit urban parks. We formed user groups based on photograph data within social media profiles, analysed their relationships with recreation behaviour at parks, as well as their effect on the spatial distribution of park visits across the city.

### Recreation behaviour through the lens of photography

Photographs as a data source for research are (1) limited by their ability to represent exactly what the human eye sees, (2) influenced by what the photographer chooses to capture (and thus exclude) in the frame, and (3) can be interpreted differently between people^40^. Photographs are thus inherently subjective, and may vary in content and style depending on the photographer. It is also important to consider that the act of sharing photographs varies between people, and can be influenced by photograph content. For example, sharing behaviour has been shown to vary based on a person’s age^41^ and geographic origin^42,43^, and tends to be higher among those who make effort to capture ‘creative’ photographs, as well as those who pose for photographs^42^. Furthermore, people may also choose to share a limited amount of content (i.e. landscapes, monuments, people, etc.) from all photographs captured^44^. Geo-tagged photographs shared online thus represent a filtered subset of a person’s experiences at a location.

Even though photographs that people capture at parks are not a holistic representation of their experiences, their content can show us the ways that people enjoy and value these locations^45^. Indeed, alongside other forms of data, photographs are becoming increasingly important for research on human behaviour and place-based experiences^40^. For instance, the act of taking photographs has been linked to human benefit^29^, and show a positive relationship with visitor happiness^46^. While we cannot know the exact intent for capturing or sharing photographs, the value given to the subject matter is made explicit when a photograph is taken and shared online^44^. A choice has to be made among all possible elements that could be captured at all possible angles, as well as among all the photographs that could be shared^44^. Analysing park users’ photograph data can thus help us understand their behaviour and preferences for outdoor recreation.

### Differences in recreation behaviour between user groups

A key comparison between park users can often be made based on their residential status. In the context of Singapore, its high level of visual greenery and worldwide reputation as a ‘Garden City’ is a likely explanation for tourists’ focus on the city and its various forms of greenery^47,48^ (Fig. 6). The look and experience of nature in the tropics are vastly different compared to other climate zones^49^, which may attract interest from those living in temperate regions. On the other hand, locals are expected to have more opportunities to take photographs at parks (Fig. 4), have better knowledge of local wildlife, and use parks for recreation (Figs. 6 and 7). Coastal areas in particular are popular locations for recreation, accessible from inland locations, and often contain many facilities and services^50^ which may have contributed to more photographs that contain ‘water/sky views and activities’ (Fig. 6). Indeed, with the exception of several major tourist hotspots^51^, spatial mapping based on users’ residential status show considerable popularity of coastal parks among locals (Fig. 5a).

Comparisons between user-group axes generated from photograph content can provide useful information when other forms of data in user profiles are scarce. Despite greater diversity in the content of favourited photographs, both uploaded and favourited photographs on Flickr produced relatively similar axes for their first two principal components—‘Landscapes–People’ and ‘Wildlife–City’ (Fig. 3). Significant relationships were observed between these axes of photograph content and photography behaviour at parks (Figs. 4 and 7), and the direction of these relationships corresponded to general expectations about preferences for nature-based recreation. For example, park users with more (uploaded and favourited) photographs of ‘Wildlife’ than the ‘City’ in their profiles tended to have more photographs captured at parks (Fig. 4), especially those of ‘nature’ (Fig. 7). When park visits were mapped according to this user-group axis, less urbanised regions with forests and nature reserves were more popular (Fig. 5c and Supplementary Fig. S10b). The high consistency in photography behaviour for this user-group axis suggests that favourited photographs of ‘Wildlife’ and the ‘City’ may be used as an indicator of park choice, even in the absence of photograph uploads. On the other hand, the spatial pattern of visits between uploaded and favourited photographs for the user-group axis ‘Landscapes–People’ was less clear (Fig. 5b and Supplementary Fig. S10a). This may be because such photograph content has a smaller impact on park visits in Singapore, or that its relationship with park visits is moderated by other physical (e.g. landscape beauty, social spaces, etc.) or behavioural (e.g. sharing motivations, online activity, etc.) factors.

### Practical implications

Quantifying behavioural patterns of park users can have implications for the planning and management of green spaces to cater to the recreational needs of city dwellers. Our study shows that photograph data in social media profiles—especially their geo-location and those of wildlife and the city—can help us form a better understanding of public interest in visits to nature areas, as well as tourist and local hotspots. Spatial variation in park visits based on users’ residential status (Fig. 5a) has revealed opportunities to promote local biodiversity at nature reserves and offshore islands amongst tourists, particularly at parks that are farther away from the city centre. Conversely, high concentration of visits by locals at specific parks can prompt further studies into what makes these places so popular among the local population. With respect to the relative amount of ‘Wildlife’ to ‘City’ photographs in user profiles, our study showed that less-manicured parks attracted users with more ‘Wildlife’ photographs (Fig. 5c). On the other hand, little to no skew observed at major attractions such as East Coast Park, Gardens by the Bay, and Sentosa Island (Fig. 5) suggests that these places are more inclusive toward different types of users. If the home locations and photograph data of local residents are available, there is potential to infer regional demand for different types of parks (i.e. naturalistic, manicured), and to assess whether such demand translates into actual visits by locals^52^.

The content of photographs captured within parks also provide an indication of park usage and enjoyment. Amongst the categories of park photographs, those that contain ‘nature’, ‘recreation’ and ‘water/sky views and activities’ were highly influenced by other photograph data in social media profiles, and differed significantly between tourists and locals (Fig. 7). A focus on such content captured at parks can have the potential to differentiate between different groups of park users, for instance, in relation to their preferences for biodiversity, recreational activities, and landscape appreciation at parks. For instance, Hausmann *et al*.^30^ examined preferences for subcategories of biodiversity derived from social media, and found greater fluctuation in preferences for charismatic biodiversity groups across social media platforms. Such biases across social media users may be captured by considering other photograph content within their profiles, as we have demonstrated in our study.

### Limitations and future developments

Interpretation of social media content offers a rapid way to understand usage patterns at parks, and is especially valuable in the absence of other data sources. However, there are limitations to consider, such as the representativeness of the sampled population, privacy concerns, data quality, as well as differences across social media platforms^53^. In our study, we performed repeated random sampling of photographs within each user’s profile (Supplementary Fig. S9), instead of selecting ‘active’ users with at least one photograph in each category^30^. This provides a more robust representation of frequencies across the photograph categories, and avoids bias toward any one user. However, the under-representation of certain groups of people, such as the elderly and technologically-disconnected, is still a cause for concern. These groups of people are often hard to reach even with conventional approaches. The growing use of ‘big’ data and models in society demands greater transparency and consideration for equity and fairness, to avoid discriminatory outputs at a systemic level^54^. Therefore, even though the use of Flickr is less affected by age and income level^30^ and the majority of Singaporeans use social media^55^, we stress that our findings are more relevant to park users who are relatively younger and technologically-savvy. Different data sources often represent varying aspects of the same location (i.e. sense of place, topographical characteristics)^56^, and sole reliance on a single platform may result in a biased perspective of recreation at parks. Studies of other social media platforms and comparisons with on-site surveys will help improve the generalisability of our findings.

While our study has uncovered behavioural patterns present among different groups of park users, these many not necessarily imply causal relationships. Future research can develop the mechanisms behind the way different groups of users value and use parks^57–59^, and to validate these links amongst park users. Links between photograph content and human well-being can also be examined, for example, by integrating both social media and survey data for canonical variate analysis^60^. There are also opportunities to examine relationships between photograph data and other indicators related to park demand such as people’s environmental attitudes^58^, ecological knowledge^61^ and nature-relatedness^62–64^, which are rarely available. Indeed, the integration of social media data into existing assessment frameworks remain key to their usefulness in park planning and management. Since multiple factors can influence the demand, supply and final provision of recreational services, a ‘systems approach’ that identifies important components and their interrelationships can help construct assessment frameworks that are useful for park planning and management^11^.

Finally, methodological improvements to the analysis of social media data can be explored. For instance, inductive approaches offered by automation can help reduce subjective interpretation often associated with manual classification of photographs^2^, and streamline the classification process as datasets grow in size. Recent work has developed methods to deal with overlapping photograph categories by weighing keywords based on probability values, and reduced the computational load by first extracting important keywords^31^. To estimate visitors’ home locations, studies have compared the performance of different measures derived from social media data, and their precision across various spatial scales^14,35^. Lastly, since photograph data often include time-based information, there is also potential to explore temporal variation in visits amongst different groups of users, and its relevance to park management or crowd control^4^.

In conclusion, our study of tropical Singapore showed that a user-focused approach to understanding recreation uncovered distinct patterns in the number and types of photographs people capture at parks. Specifically, users’ residential status and the relative amounts of ‘Wildlife–City’ and ‘Landscapes–People’ photographs in online profiles showed strong relationships with park photography. At parks, photographs related to ‘nature’, ‘recreation’ and ‘water/sky views and activities’ showed significant variations between different groups of park users. Parks were also assessed according to the kinds of users they attracted; the spatial distribution of user visits across the city corresponded to general expectations, including hotspots among tourists and locals, as well as user preferences for wildlife and naturalistic landscapes. Future work on outdoor recreation should consider the bias across different groups of park users, and those inherent within online sources of data. These will contribute toward a context-dependent understanding of human–nature interactions, and help inform the planning and management of public green spaces for the benefit of all users.

## Methods

### Study area and scope

The tropical city-state of Singapore is suitable for analyses of social media data because it consistently ranks as one of the most connected mobile consumer markets^65^ and over 70%—more than double the global average—of its population use social media^55^. Urban greening has been a key component in the city’s development approach, and there is a large diversity of green spaces island-wide, ranging from manicured parks to more naturalistic landscapes^47^. The official shape files of parks were obtained from the public data repository^66^, and edited in the software ARCGIS^67^. Nature trails in reserve areas were included, while areas inaccessible to the public were excluded. All subsequent analyses were run using the software R 3.4.3^68^.

### Photography sharing platform

Different social media platforms offer API search parameters that limit the types of content available for download, and thus their usefulness in research^69^. For photograph data, comparisons between platforms such as Panoramio, Flickr, and Instagram have been made^30,53^. While user profiles and sharing behaviour between platforms tend to be different, strong correlations have been observed for measures such as visitor counts and the geo-location of social media posts^4,18^. Studies have shown that Flickr is a reliable source of geographic content, particularly for user behaviour, because geotagging photographs is an additional rather than a primary function^70^.

### Flickr data pipeline

Photographs located within park polygons were extracted using the Flickr API; 317 out of 918 polygons had at least 1 photograph taken within their boundaries. Photograph metadata were used to identify Flickr users who visited the parks (Fig. 1a; details in Supplementary Fig. S7). The residential status of each user was assigned if it was listed on their profile, and was otherwise defined as the country where most of the users’ public photographs were taken (Fig. 1c).

The Google Cloud Vision API was used to interpret photograph content, by assigning keywords to images using a machine learning algorithm^71^ (Fig. 1b; details in Supplementary Information). The ‘RoogleVision’ package was used to access the API^72^, returning up to ten keywords per image. Hierarchical cluster analysis has the flexibility to be used on many types of data^73^, and was used to classify photographs based on their assigned keywords^21,53^ (Fig. 1b). Our approach calculates the distance (and thus similarity) between every unique pair of photographs, based on the proportion of keywords they do not share with each other, and classified photographs by maximizing the variance between clusters^21^. All keywords were converted into unique binary variables, and the distance matrix was generated based on the Jaccard distance^74^. Clustering of this distance matrix was performed using Ward’s distance^21^. To determine the appropriate number of clusters, 10% of the dataset was randomly sampled; the average difference between within- and between-cluster variation was determined across an increasing number of clusters. The L-Method was used to find the ‘knee’ of the evaluation graph by increasing the number of points assessed iteratively, starting at the cut-off value of 20; the knee was located when there was a roughly balanced number of points on either side^75^ (details in Supplementary Fig. S1). The most commonly-occurring keywords within each cluster were used to subjectively assign a name to each of the resulting photograph categories (Supplementary Figs. S2 to S4). To improve classification accuracy and the interpretability of subsequent models, related categories were aggregated after performing accuracy assessments.

### Accuracy assessments

A random sample of 1,000 park photographs was visually examined to determine whether they were accurately located within parks. Those that were obviously not taken within parks were marked as inaccurate (e.g. advertisements, etc.). The error rate was 1.1%. To assess the photograph cluster classification accuracy, a stratified random sample of photographs was mixed and manually classified into the given categories by an observer^21^. The resulting confusion matrices were used to identify categories that had a high chance of mutual misclassification, as well as those with a broad mixture of content (Supplementary Tables S1 to S3).

### Statistical analyses

To form user groups based on photograph content, robust principal component analyses was performed on the types of public and favourited photograph categories within user profiles (Figs. 1c and 3). The package ‘robCompositions’^76^ was used. Isometric log-ratio (ilr) transformation was applied; to avoid log-transformation of zero values, compositional data were converted to percentages, and a fixed value of one was added across all components^77^. The resulting loadings and scores were back-transformed to the centred log-ratio (clr) transformation space (Fig. 3).

The effect of different user groups on recreation behaviour was examined (Fig. 1c). Regression models included as predictor variables: (1) users’ residential status, as well as the principal components for the types of (2) public and (3) favourited photograph categories within user profiles. Users with favourited photographs and at least ten public photographs were analysed. Model fit was assessed by plotting the residuals against fitted values. All regressor variables were scaled, and step-wise model selection was performed based on the Akaike information criterion (AIC) and p-values for each regressor. The variance inflation factor (VIF) was used to check for multicollinearity of predictors.

The frequency of park photography for different user groups was analysed using generalised linear regression (Fig. 4). In the regression model, counts of park photographs were offset by the total upload count in each users’ profile, and over-dispersion was accounted for using the negative binomial model (n = 3,616). To analyse the effect of user groups on the types of park photographs captured (Fig. 7), those with at least five geo-tagged park photographs were analysed (n = 1,177). Dirichlet regression was used to analyse the composition of park photographs as a dependent variable (Supplementary Fig. S8), using the package ‘DirichletReg’^37^. To improve model fit and interpretability, the composition variable was a consolidation of the ten park photograph categories into five well-defined categories (Fig. 2); the category GTB was not included in this analysis owing to its broad mixture of miscellaneous content. Compositional data was transformed to address extreme values, and normalised as the composition does not sum up to 1; the ‘common’ parameterisation was used to fit the model^37^.

To calculate the frequency distribution across the ten categories of park photographs, the bias toward users with more photographs was addressed by randomly selecting one photograph per user (Fig. 6). The mean frequency across 50 samples was calculated for each photograph category, as sensitivity analysis showed that variation in the frequency distribution remained low despite increasing the number sampling repetitions (Supplementary Fig. S9). Chi-squared tests and the standardized mean-difference effect size (*d*) were used to examine differences between the proportions of the park photograph categories captured by locals and tourists.

### Spatial distribution of park users

Measures of user group variation were derived for each of the park polygons that contained geo-located photographs (Fig. 5 and Supplementary Fig. S10). To measure the relative popularity of parks based on users’ residential status, the difference between counts of tourists and local users was calculated (317 parks, 4,674 users). For user groups that were based on photograph content, the mean value of the principal component variable across all unique users was calculated for each park; parks with at least three users were mapped (177 parks, 1,177 users).

## Supplementary information

Supplementary Information.

## Data Availability

The R code for photograph classification is available in the GitHub repository, https://github.com/xp-song/photo-classify.
