# Supplementary material for: Using social media user attributes to understand human–environment interactions at urban parks
Source: Sci Rep. 2020 Jan 21;10:808. doi: 10.1038/s41598-020-57864-4 (PMC6972848; doi:10.1038/s41598-020-57864-4)
Supplement: Supplementary file 1 — Supplementary Information. [file 41598_2020_57864_MOESM1_ESM.pdf]

Using social media user attributes to understand human–environment interactions at urban parks

Xiao Ping SONG [a,b,*], Daniel R. RICHARDS [b], Puay Yok TAN [a]

[a] Department of Architecture, National University of Singapore, 4 Architecture Drive, 117566, Singapore
[b] ETH Zurich, Future Cities Laboratory, Singapore-ETH Centre, 1 Create Way, CREATE Tower, #06-01, 138602, Singapore

* Corresponding author
E-mail address: xp.song@u.nus.edu

Supplementary Information

Photograph keyword generation

The Cloud Vision technology developed by Google allows relevant information to be extracted from photographs such as keyword labels, object detection, facial expressions, and types of explicit content. Pre-trained machine learning models can be accessed through Representational State Transfer (REST) and Remote-Procedure-Call (RPC) Application Programming Interfaces (APIs). Google offers a more customisable option called AutoML Vision, which allows you to train their model with your own dataset. More information on Google Cloud Vision can be found at https://cloud.google.com/vision/docs/.

The 'RoogleVision' package is an R package that provides access to the API, and allows up to ten keywords to be returned per image (at the time of writing). More information about the installation and use of the package can be found at https://github.com/cloudyr/RoogleVision.

Photograph classification

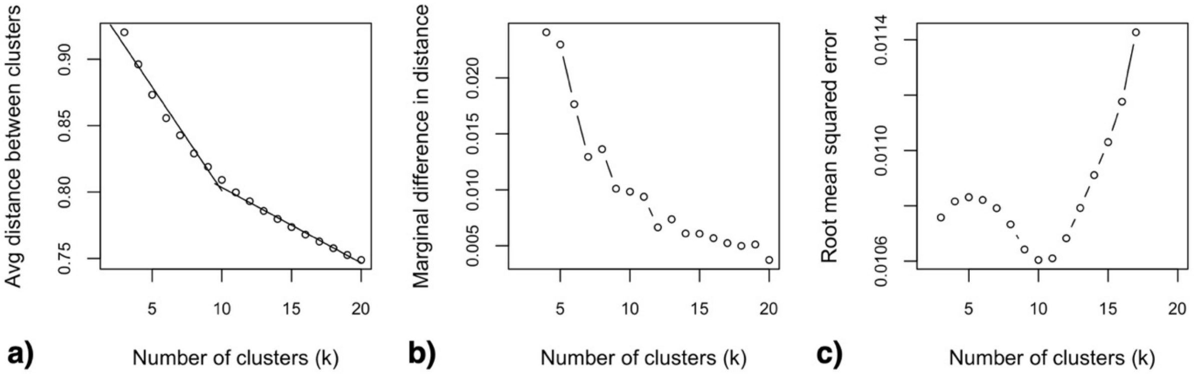

Figure S1. Park photographs were divided into ten clusters. Across an increasing number of clusters, the graphs show the (a) average distance between clusters; (b) marginal difference in distance; and (c) total root mean squared error (RMSE) of possible pairs of best-fit lines. The ‘knee’ in (a) was derived by fitting the pair of best-fit lines with the lowest RMSE. The same method was used to determine the number of clusters for users’ public and favourited photographs.

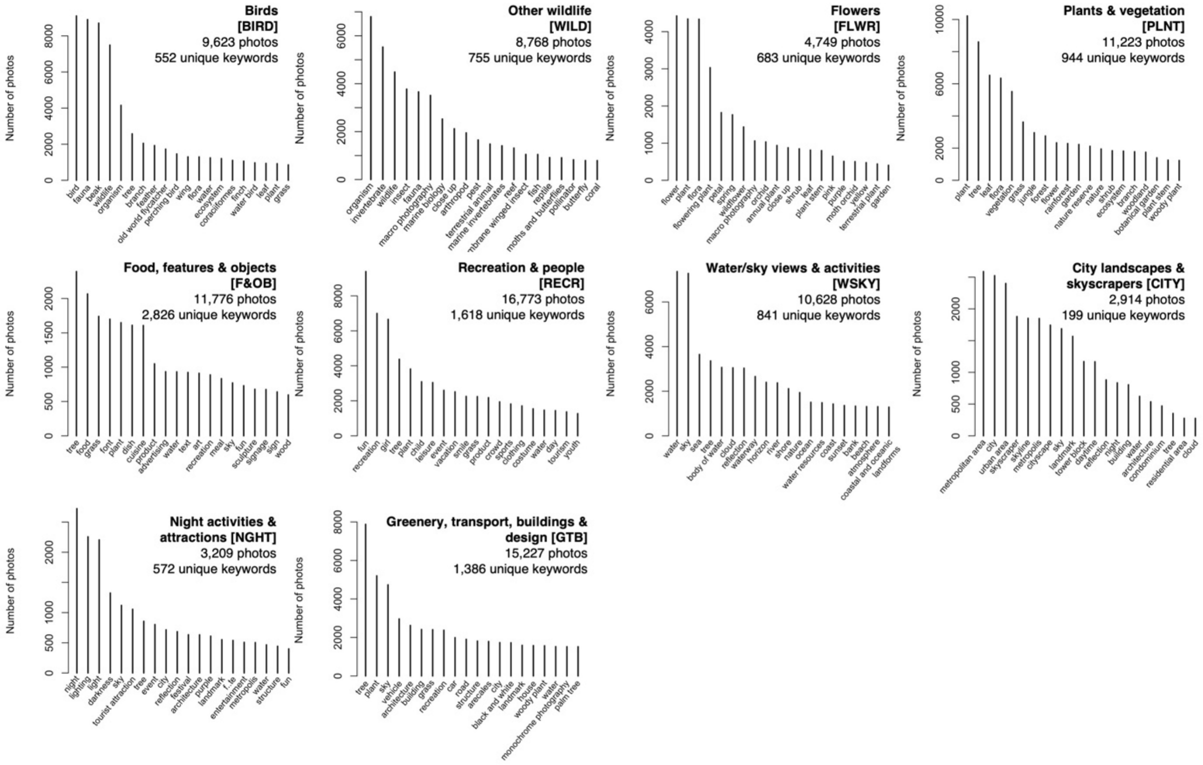

Figure S2. The frequency of the top 20 keywords generated in each category of park photographs.

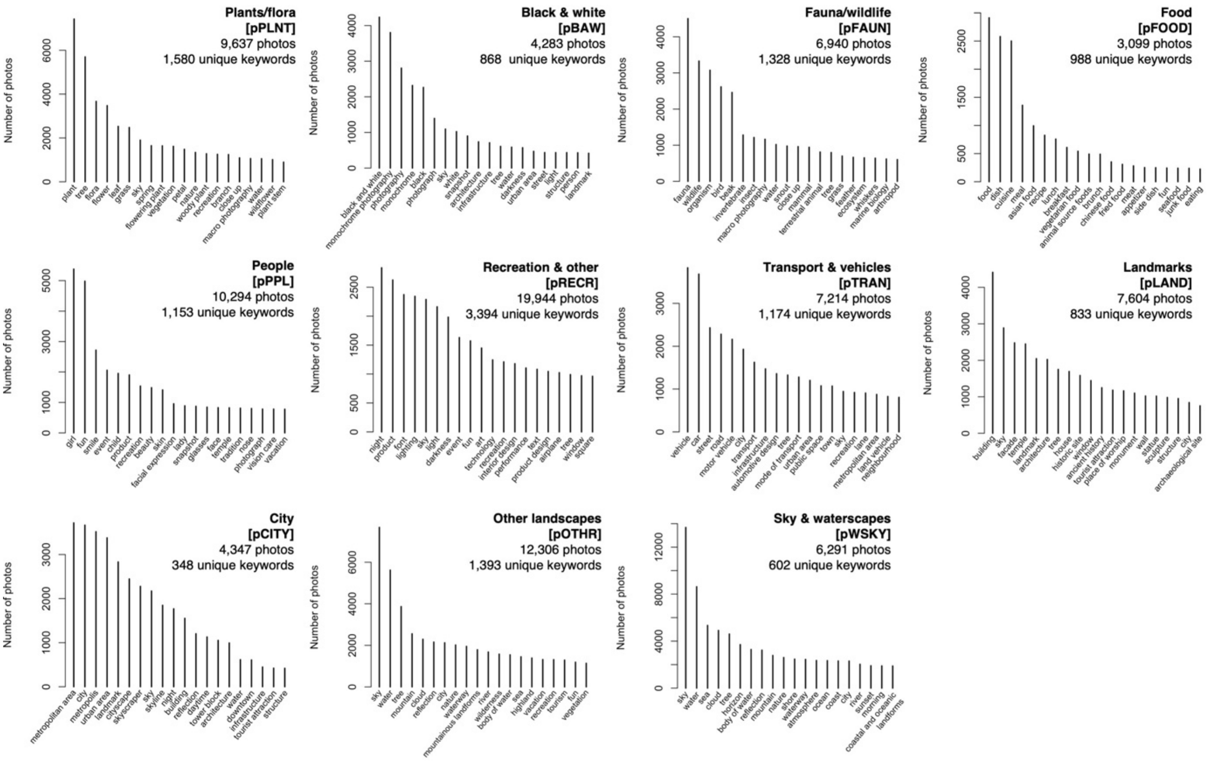

Figure S3. The frequency of the top 20 keywords generated in each category of public photographs.

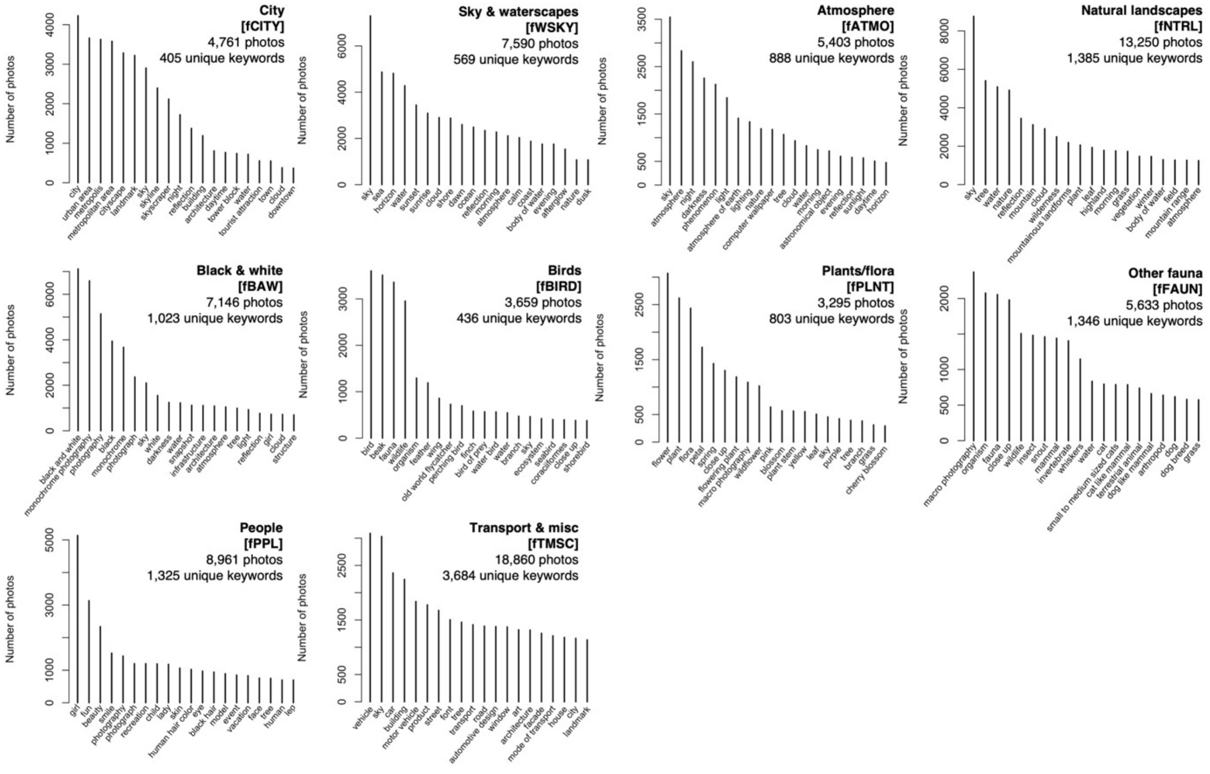

Figure S4. The frequency of the top 20 keywords generated in each category of favourited photographs.

Table S1. Confusion matrix showing the classification accuracy for park photographs. The overall accuracy was 74.49%, and the weighted Kappa value was 0.78. Photographs that were obviously not taken within parks were excluded. Superscripts denote the categories aggregated for regression analyses: [A] NATURE, [B] RECREATION.

	BIRD	WILD	FLWR	PLNT	F&OB	RECR	WSKY	CITY	NGHT	GTB	Balanced accuracy (%)
**BIRD** [A]	90	5	0	1	2	0	0	0	0	1	95.1
**WILD** [A]	1	90	0	1	6	1	0	0	0	0	94.3
**FLWR** [A]	2	6	80	7	1	1	0	0	1	0	90.5
**PLNT** [A]	2	4	6	56	4	10	0	0	0	18	76.5
**F&OB** [B]	0	1	0	4	62	7	2	1	2	15	81.2
**RECR** [B]	0	0	0	0	3	87	0	0	7	1	91.8
**WSKY**	1	2	0	7	2	2	75	2	3	4	87.1
**CITY**	0	0	0	0	0	2	12	61	8	15	80.6
**NGHT**	0	0	0	0	6	7	0	4	77	6	87.1
**GTB**	0	1	0	7	8	15	7	3	3	52	73.7

Table S2. Confusion matrix showing the classification accuracy for public photographs. The overall accuracy was 65.5%, and the weighted Kappa value was 0.73. Superscripts denote aggregated categories that were used for principal component analysis to form user groups: [A] Miscellaneous (pMISC).

	pPLNT	pBAW	pFAUN	pFOOD	pPPL	pRECR	pTRAN	pLAND	pCITY	pOTHR	pWSKY	Balanced accuracy (%)
**pPLNT**	37	0	2	0	8	4	0	3	1	0	1	81.9
**pBAW** [A]	1	44	0	0	1	8	1	0	1	0	0	88.9
**pFAUN**	2	0	47	0	0	1	0	0	0	0	0	96.6
**pFOOD**	0	0	0	46	5	1	0	0	0	0	0	93.7
**pPPL**	0	0	0	0	46	2	1	0	0	0	0	93.2
**pRECR** [A]	1	0	1	4	12	25	7	2	1	1	0	68.2
**pTRAN** [A]	4	1	0	2	8	6	21	2	6	3	2	67.8
**pLAND** [A]	0	0	0	0	1	17	0	23	9	2	1	69.6
**pCITY**	1	2	0	0	0	1	3	5	46	0	1	86.6
**pOTHR** [A]	3	1	0	0	6	11	2	9	6	15	6	61.6
**pWSKY**	0	0	1	0	0	3	0	2	2	6	43	86.7

Table S3. Confusion matrix showing the classification accuracy for favourited photographs. The overall accuracy was 69%, and the weighted Kappa value was 0.68. Superscripts denote aggregated categories that were used for principal component analysis to form user groups: [A] Water, sky, and landscapes (fWSLAND); [B] Miscellaneous (fMISC).

	fCITY	fWSKY	fATMO	fNTRL	fBAW	fBIRD	fPLNT	fFAUN	fPPL	fTMSC	Balanced accuracy (%)
**fCITY**	37	5	3	0	2	0	0	0	1	6	83.3
**fWSKY** [A]	1	29	3	9	0	2	0	0	2	2	77.7
**fATMO** [B]	1	4	13	1	2	0	2	1	5	15	64.1
**fNTRL** [A]	5	13	0	14	0	0	5	2	4	6	63.1
**fBAW** [B]	0	0	0	0	41	0	2	0	1	3	92.8
**fBIRD**	0	0	0	0	0	57	0	0	0	0	99.6
**fPLNT**	0	0	0	1	0	0	46	1	1	2	93.4
**fFAUN**	0	0	0	0	0	2	6	33	3	7	81.9
**fPPL**	0	1	0	0	1	0	0	0	42	12	84.9
**fTMSC** [B]	2	0	0	0	2	0	0	0	6	33	82.6

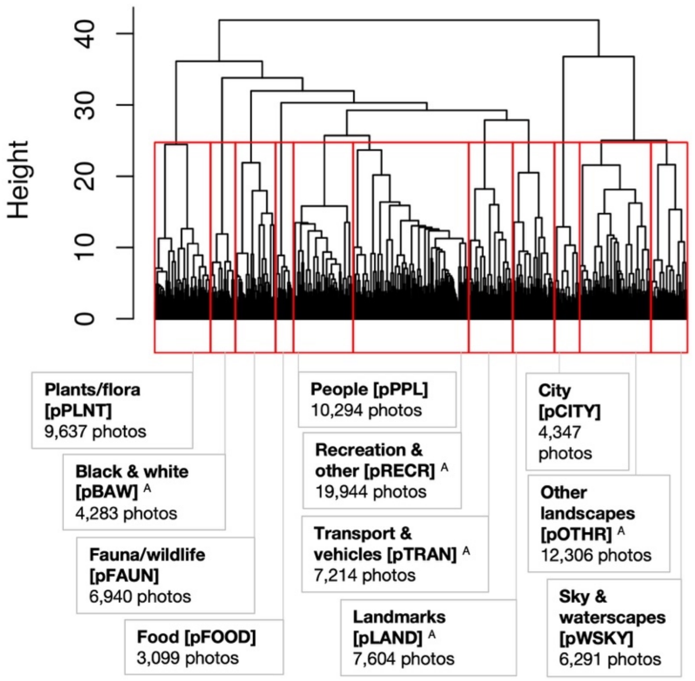

Figure S5. 11 categories of park users' public photographs after hierarchical clustering. The abbreviation for each category name is shown in square brackets. Superscripts denote aggregated categories that were when used for principal component analysis of compositional data: [A] Miscellaneous (pMISC) (more details in Supplementary Table S2).

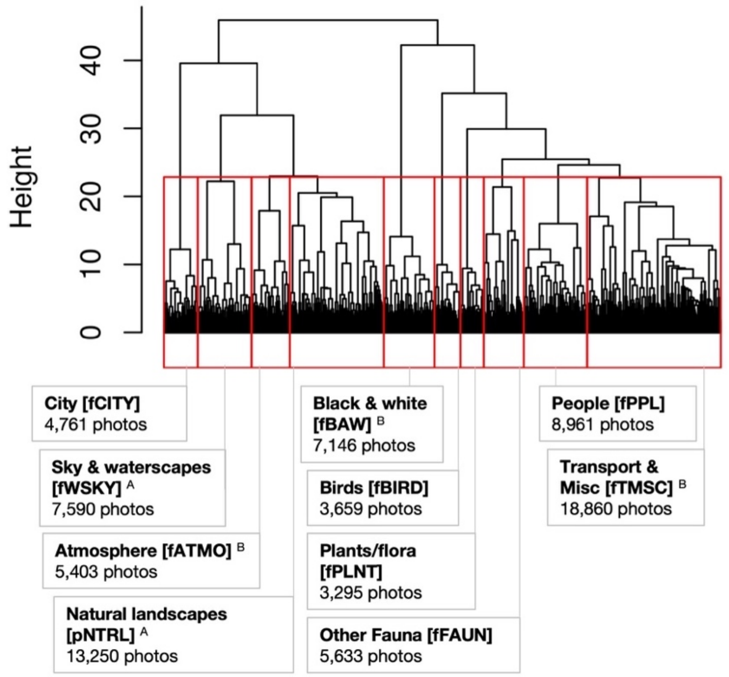

Figure S6. 10 categories of park users' favourited photographs after hierarchical clustering. The abbreviation for each category name is shown in square brackets. Superscripts denote aggregated categories that were when used for principal component analysis of compositional data: [A] Water, sky and landscapes (fWSLAND); [B] Miscellaneous (fMISC) (more details in Supplementary Table S3).

Park photographs: Summary statistics

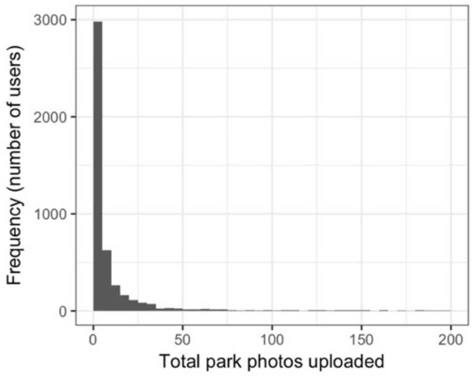

Figure S7. Histogram of users' total uploads within park polygons (bin width = 5). Users with up to 200 relevant uploads are shown. 75 out of the 4,674 users are not shown. Ten users had more than 1000 relevant uploads.

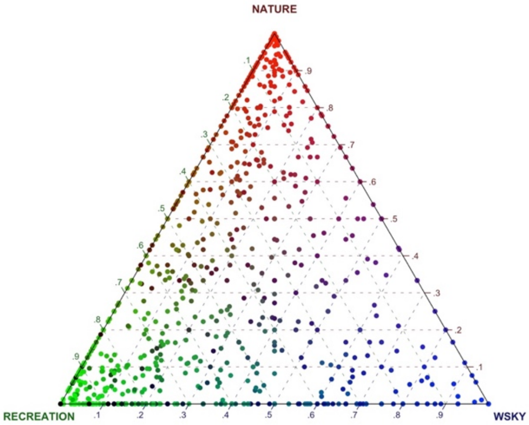

Figure S8. Distribution of users based on the types of park photographs within social media profiles. Only three out of five photograph categories are shown in this 2-D visualisation. All five categories were analysed as a composition (dependent) variable in regression analysis.

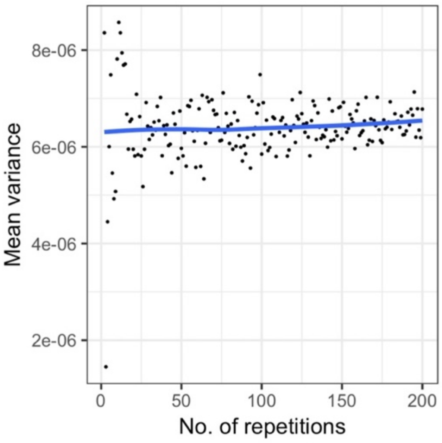

Figure S9. Sensitivity analysis for the variance in the frequency distribution across the ten categories of park photographs, based on the number of times a photograph was sampled per Flickr user. The scatterplot shows the change in mean variance across an increasing number of times the sampling was repeated.

Spatial distribution of park users

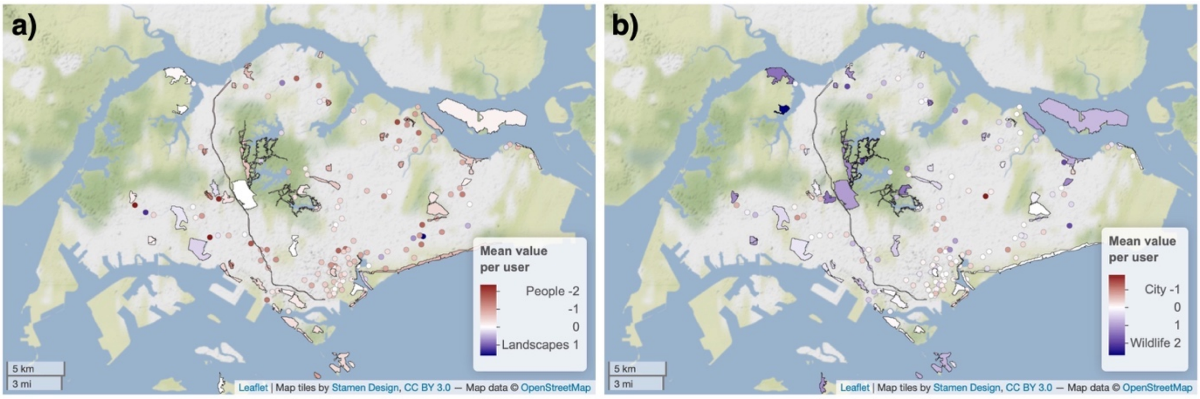

Figure S10. Variation across parks in Singapore according to the kinds of social media users they attract. User groups were based on the content of publicly uploaded photographs using the principal component axes (a) Landscapes–People and (b) Wildlife–City. Parks less than 0.1 km^2 are shown as circles. Data sources for base maps: Stamen Design; OpenStreetMap.
